# Semi-natural habitats in the European boreal region: Caught in the socio-ecological extinction vortex?

**DOI:** 10.1007/s13280-022-01705-3

**Published:** 2022-02-12

**Authors:** Irina Herzon, Kaisa J. Raatikainen, Aveliina Helm, Solvita Rūsiņa, Sølvi Wehn, Ove Eriksson

**Affiliations:** 1 Department of Agricultural Sciences, University of Helsinki, P.O. Box 27, 00014 Helsinki, Finland; 2 Helsinki Institute of Sustainability Science, HELSUS, University of Helsinki, P. O. 20 Box 65, 00014 Helsinki, Finland; 3 Department of Biological and Environmental Science, School of Resource Wisdom, University of Jyvaskyla, P.O.Box 35, 40014 Jyvaskyla, Finland; 4 Institute of Ecology and Earth Sciences, University of Tartu, Lai 40, 51005 Tartu, Estonia; 5 Faculty of Geography and Earth Sciences, University of Latvia, Jelgavas iela 1, 1004 Riga, Latvia; 6 Milticonsult Renewable Energy, Sluppenveien 15, 7037 Trondheim, Norway; 7 Department of Ecology, Environment and Plant Sciences, Stockholm University, 106 91, Stockholm, Sweden

**Keywords:** Agriculture, Biodiversity, Conservation, Farmland, Socio-ecological systems

## Abstract

We propose to consider semi-natural habitats—hotspots for biodiversity—being caught in a *socio-ecological extinction vortex*, similar to the phenomenon described for species threatened with extinction. These habitats are essentially socioecological systems, in which socioeconomic drivers are interlinked with ecological processes. We identify four highly interlinked and mutually reinforcing socio-economic processes, pertaining to the importance of semi-natural habitats for (i) agricultural production, (ii) policy, research and development; (iii) vocational education in the fields of agricultural sciences and (iv) public’s experiences with semi-natural habitats. Evidence from six countries in the boreal region demonstrates that recent slowing down or even reversal of two processes are insufficient to stop the extinction vortex phenomenon. We suggest research directions to ascertain the phenomenon, monitor its development and develop proactive actions to weaken the vortex. It is highly plausible that interventions directed at most, if not all, of the key vortex processes are needed to reverse the overall deteriorating trends of a socio-ecological system.

## Introduction

Most of biodiversity associated with agricultural landscapes on the European continent resides in so-called semi-natural habitats, so much that areas with high semi-natural vegetation cover are called High Nature Value (HNV) farmlands (Paracchini et al. 2008). Semi-natural habitats, such as meadows and wood-pastures, traditionally functioned as a vital endogenous source of fodder for livestock and provided nutrients to crop fields through manure harvesting, forming a complex system of nutrient and species flows across landscapes (Eriksson et al. 2021). However, the role of semi-natural vegetation became obsolete in modern farming systems, in which crop production derives substantial resources from fossil energy, mineral fertilisers, and other non-renewables. Intensive systems of crop and ruminant production, the latter often forgoing grazing altogether, became the cornerstone of agricultural industries during the twentieth century (e.g., van den Pol-van Dasselaar et al. 2020; Eriksson et al. 2021). Only in marginal agricultural regions in Europe do semi-natural habitats remain an important resource for farming systems dubbed HNV farming systems (Keenleyside et al. 2014). Elsewhere, remnants of semi-natural vegetation are managed increasingly for conservation rather than as part of farming systems (*ibid*). With a considerable decline of the historical coverage of the semi-natural habitats—over 90% over the past century (EEA 2016)—increasing attention has been drawn to the unique value of such habitats for achieving biodiversity conservation targets (e.g., Halada et al. 2011). Apart from their conservation value, semi-natural habitats are increasingly acknowledged for other public benefits such as scenic values, cultural heritage, carbon sequestration, water retention and as reservoirs of genetic diversity (Torralba et al. 2018; Bengtsson et al. 2019).

Despite the increased understanding of threats and conservation needs (Squires et al. 2018), the extent and quality of semi-natural habitats continue to decline in Europe (EEA 2020). The existence of semi-natural habitats depends on active management by some actor in society. This role has traditionally been filled by the farmer, and commonly still is, but other actors (e.g., conservation authorities, NGOs, foundations) take charge. The continuation of management requires that semi-natural habitats represent meaningful economic and cultural assets and have presence in the mindscape of society (Agnoletti and Rotherham 2015). Unlike natural ecosystems, species and communities of semi-natural habitats are therefore directly under the influence of both natural processes and human management, and are thus shaped by socio-economic drivers. The failure to halt the decline may result from an inherently complex character of a socio-ecological system (Fischer et al. 2017), and challenges in communicating this complexity across disciplines and among stakeholders (Raatikainen 2018; Funk et al. 2020). Although many recent studies (e.g., Wehn et al. 2018; Lomba et al. 2019; Veidemane et al. 2019) have shed light on interactions of drivers in the context of semi-natural habitat management, we still lack a simple yet informative framework to guide research on the semi-natural habitats as complex socio-ecological systems.

We propose to upscale a well-established concept in conservation biology of the species *extinction vortex* to habitat level. Originally, the extinction vortex described the additive threats that declining populations face, originating from both biotic and/or abiotic factors (Gilpin and Soulé 1986). The central idea is that each of the negative processes reinforces another or several other negative processes, which together create a vicious cycle (extinction vortex), making an already small population even smaller and increasingly vulnerable to stochastic events (Gilpin and Soulé 1986). These feedbacks eventually lead the population to extinction. In this paper, we extend the extinction vortex framework by integrating social drivers and feedbacks that affect a habitat through impeding its management, which is needed for habitat sustenance. Once a habitat starts to decline and becomes increasingly rare, the feedback processes start operating in mutually reinforcing ways, thus making the habitat prone to disappearance both from land- and mindscapes. We scrutinize the evidence pertaining to the potential feedback processes available for six countries in the North European boreal region: Norway, Sweden, Finland, Estonia, Latvia and Lithuania. Though the case is built around semi-natural habitats, the approach could inform also other rare habitat types in need of active conservation management.

## Socio-ecological extinction vortex for semi-natural habitats

**Fig. 1:**
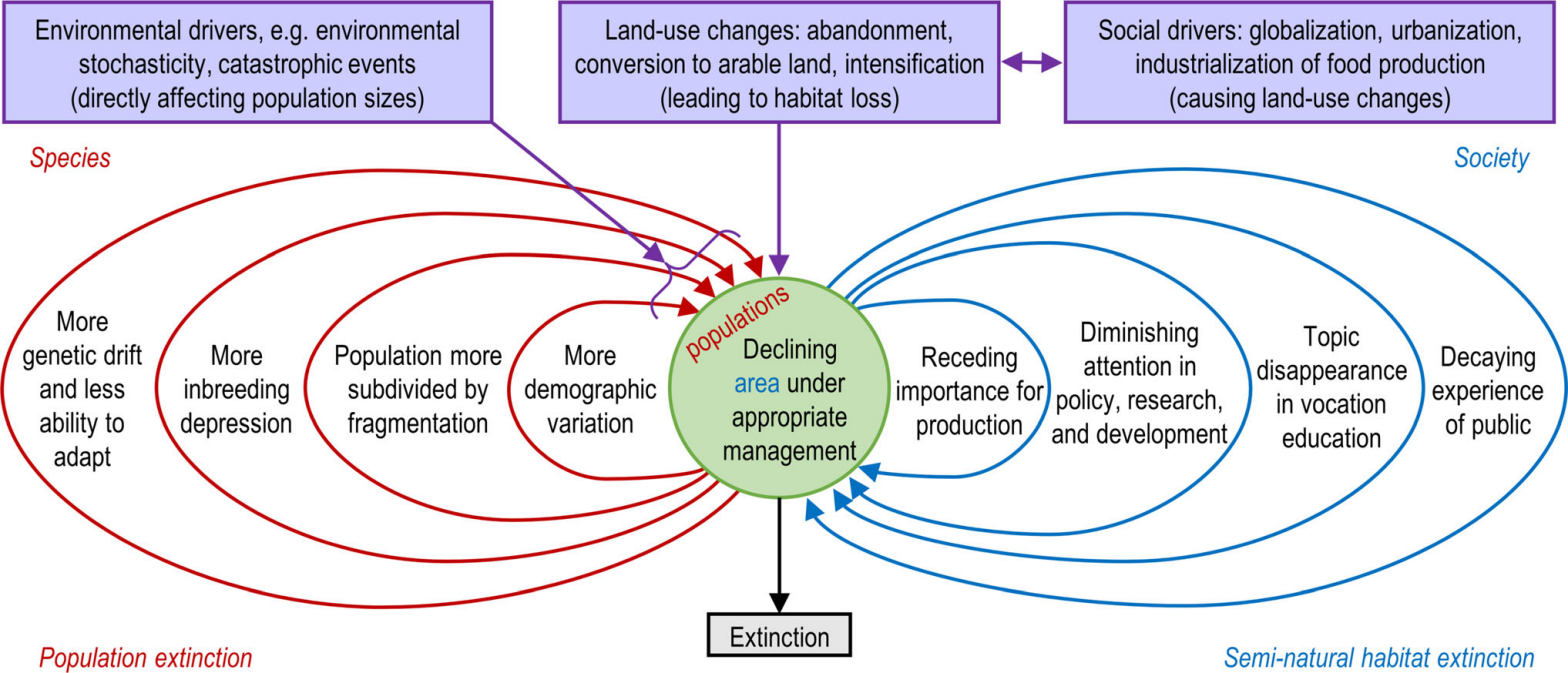
Socio-ecological extinction vortex that impacts the quantity and quality of semi-natural habitats through their adequate management includes both ecological (red feedback loops, leading to extinction of species populations) and societal processes (blue feedback loops, leading to habitat extinction). The onset of the socio-ecological extinction vortex is determined by social drivers of land-use changes (boxes on top of the figure). The feedback loops in the vortex exert negative and mutually reinforcing effects on a focal habitat wherein the species populations are nested, and are in turn affected by the deteriorated state of the habitat. Adapted from Primack (2010) Fig. 11.14

To start with, we propose to set the focal parameter in the socio-ecological extinction vortex as “area under appropriate management”, which refers to the habitat coverage managed in ways that maintain it in a “good degree of conservation” (sensu the Habitats Directive 1992) (Fig. 1). For semi-natural habitats, management is essentially maintaining the characteristic ecological structures and functions. Defining “appropriate management” unambiguously is difficult, but some principles of it are well recognised (e.g., Lindgren and Cousins 2017). Firstly, it is a management regime under which the diverse species community has been formed and maintained in a dynamic manner through centuries. For example, areas maintained by haymaking may undergo considerable community shifts if grazed (e.g., Tälle et al. 2016). Secondly, the management intensity should be compatible with the disturbance tolerance of the characteristic species (e.g., grazing pressure, Tälle et al. 2015). Thirdly, the spatial extent of management should be adequate, as communities of small and highly fragmented patches may lose species despite the appropriate management (Cousins 2009). Appropriate management would allow the communities to be dynamically stable while preventing the extinction debt (Helm et al. 2005).

Environmental drivers, such as biotic and abiotic stochasticity and catastrophic events, in the original species extinction vortex (Primack 2010), remain important also for the socio-ecological extinction vortex (Fig. 1). These affect the populations directly through reducing their sizes, and causing the population-level feedback loops. Populations occupying a semi-natural habitat are also reduced by its area under appropriate management, which in turn is an outcome of land-use changes. Management abandonment, conversion to arable land and intensification of grassland use are key land-use changes specific to semi-natural habitat loss (Aune et al. 2018; Eriksson et al. 2021), and are driven by large-scale phenomena of globalization, urbanization (accompanied by rural depopulation) and industrialization.

The socio-ecological extinction vortex consists both of ecological and societal feedback loops, which contribute to continuous decline and deterioration of semi-natural habitats and their populations’ sizes (Fig. 1). Specific ecological processes driving the extinction vortex of small populations include demographic variation, inbreeding depression and genetic drift (Primack 2010), also on semi-natural grasslands (Honnay and Jacquemyn 2007; Picó and Van Groenendael 2007). The process are also intensified by declining population sizes. We propose four social processes that similarly reinforce each other, and both affect and are affected by the area under appropriate management: (i) receding importance of semi-natural habitats for agricultural production, (ii) diminishing attention to semi-natural habitats in policy, research and development; (iii) disappearance of the topic in vocational education in the fields of agricultural sciences and (iv) decaying experience of the public with semi-natural habitats as dependent on human management. For the rest of the paper, we call these processes ‘loops’ and retain the term ‘vortex’ for the overreaching phenomenon.

The first loop in the socio-ecological extinction vortex is a continuation of the historical shift from using semi-natural habitats as a production resource to perceiving them as non- or poorly productive land-uses. This leads to an initial loss in managed area. Once the area of semi-natural habitat under appropriate management becomes sufficiently small, the vortex phenomenon starts, that is, the decline in area and in importance for production start reinforcing each other. Because of the marginalization in food production, attention to semi-natural habitats’ relevance in policy, research and development also diminished, which is a potential second loop. During the decades of agricultural intensification, semi-natural habitats were destined for so-called amelioration through drainage, re-seeding and fertilisation (Eriksson et al. 2021), with the aim of transforming them into what was considered as productive land. Thus, this loop builds on the first one and, in time, cements its impact through a reinforcing feedback that detached semi-natural habitats from other agricultural land-uses (Raatikainen and Barron 2017).

Managing semi-natural habitats in production is particularly knowledge-intensive and requires local understanding of spatial and temporal variability in the amount and quality of biomass as pasture and hayed fodder. Once professional skills based on tradition (loop 1) and access to novel understanding, advice and innovation (loop 2) disappear, farmers may become increasingly risk avert against using semi-natural habitats in production. This leads to erosion not only of professional knowledge but also the demand for it. Therefore, we suggest that the third loop includes disappearance of vocational and higher education teaching, that is, knowledge transfer, about semi-natural habitats as production resources (loop 3).

According to the third loop, professional educational programmes have dropped semi-natural habitat management from their curricula as an irrelevant topic in the know-how and toolkits of actors in the agricultural sector. The educational focus shifted to ways of overcoming the environmental limitations of land to achieve high yields of crops and timber, rather than on ways of utilising diverse local resources over the whole landscape, optimising production for multiple benefits (e.g., agroforestry) and minimising external inputs. Education on semi-natural habitats also moved to the ecological disciplines, frequently separated from agricultural sciences by disciplinary and campus borders. Poor supply of knowledge on the topic (loop 3) reinforces difficulties of land-users in making use in production (loop 1) and understanding of engagement needs and opportunities in development and policy (loop 2).

The three loops described above pertain to professional spheres around the agricultural production. We propose that the socio-ecological extinction vortex advanced into the public arena once the final loop started to operate: the decreasing experiences of the general public on semi-natural habitats as tied to human management (loop 4). The disappearance of semi-natural habitats from the public mindscape follows from their decline in the everyday landscapes, as well as from the processes that further marginalize semi-natural habitat management in the society (loops 1–3). The shrinking area of semi-natural habitats and, frequently, situation in remote and inaccessible locations (e.g., Aune et al. 2018) make the habitats “invisible” and unrecognised by society. In the worst-case scenario, this may culminate in the accustomisation of the public with the loss of semi-natural habitats; the habitat rarity is considered acceptable, and people lose interest in managing, conserving, and appreciating semi-natural habitats. This change in public attitude, in turn, reinforces the previous loops of the vortex as the public does not demand professionals to maintain semi-natural habitats but may even support their further conversion into other land uses. For example, to mitigate climate change impacts, agriculturally unimproved land, which often equates with semi-natural habitats, has been dedicated to tree planting under the EU Biodiversity Strategy (Tölgyesi et al. 2021). We hypothesise that the weakened public pressure (loop 4) is reflected back in food production (loop 1), policymaking, research, and development (loop 2), and education (loop 3); all of which develop a blind spot for semi-natural habitats. All of these started operating from the onset of land-use change, probably at different times and with varied levels of concurrency, depending on the region.

The fundamental feature of the socio-ecological extinction vortex is that the processes within it reinforce each other, as demonstrated here and by Primack (2010). The feedbacks also intensify with the decline in a habitat area under appropriate management, which causes declines in species populations, thus linking the ecological and social sides of the vortex. Declines in the area of semi-natural habitats and in habitat patch sizes at each farm, and their fragmentation across the landscape make the ongoing use in production and/or retake into production more expensive and difficult. This is due to the increased machinery sizes designed for large fields with even surfaces and the increase in herd and animal sizes. Complications faced by farmers when using small fragments for production lead to further semi-natural habitat abandonment, and decline in populations of already threatened species. As producers’ needs in the know-how of managing such areas diminishes, vocational education, agricultural policy and mainstream research in animal husbandry and fodder production would receive a receding demand for focusing on such areas. An example comes from the drastic declines in grazing dairy cattle, which caused a concern for “the loss of grazing skills” (van den Pol-van Dasselaar et al. 2020).

## Evidence for the socio-ecological extinction vortex

Below, we discuss the scope of evidence, and gaps in it, for the *socio-ecological extinction vortex* for semi-natural habitats in the boreal European countries. We also outline potential research directions needed for corroborating and improving the concept as a tool in reversing the extinction vortex process.

### Area under appropriate management and importance in production

Ideally, the “area under appropriate management” should be derived from observations on the ecological quality of semi-natural habitats, i.e. monitoring of species populations and community compositions, and abiotic factors (Keith et al. 2013). However, reliable and consistent information on the quantity and quality of semi-natural habitats is generally lacking, especially for non-grassland types, such as grazed forest and coastal heath (Plieninger et al. 2015; Herzon et al. 2021). Estimates for managed semi-natural grasslands range within 20 000–50 000 hectares in each country, except for Sweden with ca. 300 000 hectares (Herzon et al. 2021). The remaining areas of semi-natural habitats are estimated to only be a few percent of their historic areas (Cousins et al. 2015; Hovstad et al. 2018; Lehtomaa et al. 2018).

Across boreal Europe, the species specialized into semi-natural habitats demonstrate particularly high levels of endangerment. In Norway, 55% of the assessed species with semi-natural grassland and heathland as main habitats are red-listed and 35% are threatened (classes CR, EN or VU) (Artsdatabanken 2021). In Sweden, over 20% of the assessed species in agricultural landscapes are threatened or near threatened, representing roughly a third of all red-listed species in Sweden (Eide et al. 2020). Threatened species are generally dependent on semi-natural habitats in these agricultural landscapes. In Estonia, ratios of red-listed species to all species from semi-natural grasslands range 20–86%, depending on the taxon (Pärtel et al. 2007). Of all protected species, 50% depend on semi-natural grasslands either as habitat or for feeding (Helm et al. 2020). In Latvia, semi-natural grasslands host nearly 30% of the vascular plant, invertebrate and bird species (Rūsiņa 2017). In the case of Finland, 24% of threatened species live and 40% of all extinct species lived in agricultural landscapes, largely semi-natural habitats (Hyvärinen et al. 2019), which are by far the most endangered habitat types in Finland (Lehtomaa et al. 2018).

When ruminant-based production develops towards ever greater specialisation and intensive animal rearing (e.g. zero-grazing dairy and calves fattening, grain-based beef production), the potential for semi-natural habitats to support production is increasingly lost. The management of considerable areas of semi-natural habitats is currently irrelevant for production across the region; the shares of permanent grassland managed for subsidies and not for production range from 11% (Lithuania) to 58% (Norway) (EUROSTAT 2013). In Finland, where the use of semi-natural habitats in production is marginal, continuously managing as little as 20 000 hectares proved challenging even with subsidies and restoration targets (Raatikainen et al. 2017). This phenomenon is also reported from Norway (Wehn et al. 2018). On the other hand, Sweden retained use of pastures generally and semi-natural ones in particular to a regionally exceptional level (Herzon et al. 2021). The basis for this was laid in 1988, when the Swedish parliament passed a bill that “gave Swedish cattle grazing rights”, which required all cattle to have access to pasture during the growing season (Animal Protection Index 2020). This is in contrast to Finland where the pasture area halved nationally in two last decades (Natural Resources Institute Finland 2021).

Data on fodder production volumes, added value products and farm economics related to the use of semi-natural habitats are basically non-existent in the region (Herzon et al. 2021). Products from semi-natural habitats are mostly not differentiated from those of intensive systems (three labels in the region are presented below). Other value-added benefits to the farm economy, such as recreational activities (holiday homes, green care, hobby equine activities) are poorly described, though are known to encourage semi-natural habitat management (*ibid*).

Based on the shares of current areas under management, trends, the persistence of threats and the endangerment of specialist species, we conclude that the ecological state of semi-natural habitats—reflected in the area under appropriate management—continues to deteriorate. We also witness signs in each country that the reduction in semi-natural habitat coverage and its fragmentation drives further decline in producer motivation for management. Focusing research and innovation on diversified production and its associated values in semi-natural habitats in regions heavily centred on ruminant-based production could be a way to weaken the loop. Testing to what extent the production role of semi-natural areas is related to the attention they attain in other social spheres is needed to ascertain the feedback process.

### Importance in policy, research, and development

Though agricultural policy neglected semi-natural habitats for most of the post-World War II period, conservation policies set them on the political agenda in the 1990s. The maintenance of semi-natural habitats became part of the agri-environment-climate measures (AECM) in most EU countries, and in a similar national policy in Norway (Wehn et al. 2018; Alliance Environment 2019). However, internal inconsistencies and flaws in the policies have prevented the reversal of negative trends for the “area under appropriate management”. Firstly, there is a much larger budget for farm enlargement and modernisation than the relatively small sums spent on ecological objectives (Pe’er et al. 2020). In Finland, it became impossible to apply for new contracts for the semi-natural grassland management in last years of both programming periods of 2007–2013 and 2014–2020 due to the lack of funds in the overall agricultural policy budget. A low payment level combined with occasionally excessively strict eligibility requirements are main obstacles in Estonia (Holm et al. 2019; Veidemane et al. 2019). Eligibility is thus a second obstacle. Only semi-natural grasslands within the Natura 2000 network are eligible for AECM payments in Estonia. Wooded pastures and grazed forests are mostly ineligible due to their high tree coverage. Finally, landowners are denied incentives if they are not active farmers (Raatikainen and Barron 2017).

Third, policy interventions have narrow targets. They promote single objectives at a time, such as the species richness of vascular plants, and set arbitrary restrictions such as those for tree cover on pastures (Lindborg et al. 2008), and typically focus on areas that are easy to measure and manage. Such narrowly focused preservation strategies fail to acknowledge that semi-natural habitats are essentially parts of larger farming systems and landscapes with multiple values that are based on deep and coevolved linkages between people and nature (Lindborg et al. 2008; Fischer et al. 2017). An overly strong emphasis on financial payments targeting specific farming practices further reinforces a decoupling of social and ecological subsystems (Raatikainen and Barron 2017; Wehn et al. 2018).

The introduction of related policies has encouraged research on semi-natural habitats in Europe, especially on public values and socio-economics (Torralba et al. 2018; Lomba et al. 2019; Herzon et al. 2021). This can be interpreted as a sign of increasing interest on societal feedbacks around semi-natural habitat management. However, the attention mainly comes from the ecological research community, not agricultural. This is illustrated by the abstracts submitted to the 2020 conference of the European Grassland Federation (Virkajärvi et al. 2020). Of the total 230 abstracts, only eight dealt specifically with semi-natural habitats and just half of these included production aspects. Of 113 abstracts in the sessions devoted to production aspects of grasslands, only one touched upon semi-natural grasslands. Contrastingly, in a session on Grasslands and Environment, 22 abstracts out of 62 included biodiversity aspects. Almost no research is done integrating evidence of private and public benefits along the gradient from intensively managed grasslands (agricultural science perspective) to semi-natural grasslands (conservation perspective) (Bullock et al. 2020). Thus research on semi-natural habitat is separated from that in agricultural production—a disciplinary divide evident also in higher education (see Section Vocational training and higher education).

Increasing the relevance of semi-natural habitats includes co-creation and uptake of best practices and innovative solutions, networking and governance—all typically realized through development projects. We analysed the share of EU-funded projects focusing partly or entirely on semi-natural habitats in relation to the overall numbers of projects from major funding instruments, such as the LIFE programme, the European Agricultural Fund for Rural Development (LEADER groups), the European Innovation Partnership for Agricultural productivity and Sustainability (EIP-AGRI) and the Regional Development Funds (Interreg) (Box 1). Apart from the EU, considerable national funding is channelled through these instruments because of their co-funding requirement.

During its operation in 1992–2020, the LIFE Programme funded 155 projects in the region, focusing on biodiversity issues. Out of these, 48 (31%) included semi-natural habitats among their operational priorities or as one among several focal habitats. In the Baltic States, the share of such projects falls within 40–60%, while the same shares in Finland and Sweden are 30% and 25%, respectively. Attention to semi-natural habitats sharply dropped among other programmes. Out of 36 examined LEADER projects focusing on agriculture, only two projects explicitly included semi-natural habitat management and one potentially so (https://enrd.ec.europa.eu/projects-practice_en). Of the 43 Interreg projects funded in 2014–2020 under the Nature resources category, none had relevance to farmland nature (https://www.interreg-baltic.eu/home.html). Of 47 multi-actor projects funded by the EIP-AGRI mechanism under Horizon 2020, ten with potentially relevant foci operated in the region (https://ec.europa.eu/eip/agriculture/en/eip-agri-projects/projects/multi-actor-projects). Only two projects in the region included studies or innovation cases with semi-natural habitats. Of the projects not active in the region, only three out of a total of 85 innovation cases for grasslands were for semi-natural ones. A single project explicitly focused on HNV farming.

Attention to semi-natural habitats in policy, research and development has thus been slightly increasing in the past decades. However, both in research and development, this comes mostly from conservation-oriented programmes and marginally so in the agricultural field. An example of a profound impact a production-related legislation may have is illustrated above by an animal welfare policies in Sweden. Therefore, the knowledge and governance related feedback loop (loop 2) continues to fuel the socio-ecological extinction vortex for semi-natural habitats due to its conservation-only focus. A much needed research agenda is for transdisciplinary studies on participatory governance, development of political environment with synergetic objectives, integration of rural viability, social acceptance, multiple production and public goods aspects. Evidence that the research effort on semi-natural habitat is associated with their management for multiple benefits, including production, prominence of the topic in vocational education and in public awareness would be a powerful sign of the vortex phenomenon.

### Vocational training and higher education

Little evidence is available concerning the extent to which “semi-natural habitats” or “extensive grassland” or “High Nature Value farming” are being introduced to future agricultural professionals in vocational training and higher education. Karjalainen (unpubl.) collected responses from 18 teachers and 51 students from all eight vocational training schools with agricultural study lines in Finland. Only 45% of students agreed being well-taught on a topic concerning semi-natural habitats, and mere 6% found the topic useful. This contrasted with the perceived usefulness of knowledge on biodiversity in general (37%). Also, 61% failed to describe the suitable management of a semi-natural habitat. A question on respondents’ familiarity with types of semi-natural habitats (such as “dry meadow”, “grazed forest”, and “wooded meadow”) resulted in affirmative answers from only 12–41% of students; the respective proportion among teachers was 20–51%. This indicates that specific terms for traditional types of agricultural land uses may be vanishing from modern agricultural vocabulary. As one teacher explained: "… we follow the nationally approved guidelines for qualifications. In agricultural studies, aspects… of nature management and biodiversity enhancement are not mentioned”. Teaching related to semi-natural habitats in vocational schools is left to the discretion of individual teachers according to their personal interest and knowledge, and variation among institutions seems large. No other research from the region exists, but, to our knowledge, teaching on semi-natural grasslands is mostly run within nature conservation topics rather than agricultural sciences, and is limited in scope.

The above is corroborated by a survey on teaching about HNV farmlands and farming systems, sent to over 300 higher education personnel in the agricultural, sustainable development and conservation biology studies across the EU. It returned 62 replies (20% response rate) (Herzon and Koivuranta, unpubl.). The respondents who used the HNV concept (ca 60%), did it from the perspective of ecology/biology (74%), rural development (53%) or agronomy (34%). One respondent said that teaching about HNV farmland would require “a change in the philosophy of studies in the faculty of agriculture… to teach sustainability …not only classic courses”. Overcoming such disciplinary boundaries in higher education is a slow process laden with challenges (Ng & Litzenberg, 2019), yet it is essential for teaching and researching socio-ecological systems.

In the field of education, the Erasmus Programme is the EU’s most important funding tool. Only two Erasmus projects have had an explicit focus on semi-natural habitats or conservation in agriculture (both launched in 2000 and one with a partner in the boreal region). Link to formal education was weak in most EU-funded projects reviewed in Section Importance in policy, research, and development, where most effort was given to advisory services. Only few projects explicitly worked with professional education in bridging agricultural sciences and conservation (e.g., Herzon 2018; Inno4Grass 2021).

Research across the countries with varied areas of semi-natural habitat might be particularly enlightening. It could target long-term effects professional education curricula may have on other related phenomena, such as, for example, motivations of farmers in overcoming the barriers to management for production, advisors to actively promote tools supporting such production, innovators to search for modern solutions to what is rendered traditional, and finally public awareness and consumer decision-making. For the time being, our assertion that educational emphasis is a separate feedback process in the socio-ecological extinction vortex remains speculative.

### Public experience

Only a handful of studies look at public awareness concerning semi-natural habitats (e.g., Garrido et al. 2017; Viirret et al. 2019). A Swedish survey demonstrated that wood-pastures were considered important by 60% of 1000 respondents, mainly for their biodiversity, and 40% of meat consumers were willing to pay a premium for animal products from wooded pastures (Kumm 2017). Furthermore, wood-pastures positively affected surrounding property prices. In Finland, interviews with producers with semi-natural pastures indicated that customers buying meat directly from such farms valued ‘the overall wellbeing of the animals’ but had little awareness of the differences between cultivated and semi-natural pastures (Kaljonen 2018). Producers believed it would be prohibitively difficult for them to educate their customers about the differences between the two pasture types (Kaljonen, pers. comm.). This finding is in line with evidence on profound differences in perceptions of producers and increasingly urbanized consumers on the nature of semi-natural habitats (Raatikainen and Barron 2017).

The commercial success of brands related to semi-natural (or natural) meadows would be one indication of consumer awareness. A Swedish national brand (Naturbeteskött, www.naturbete.se) became commercially successful with sufficient domestic demand (Jamieson, pers. comm.). However, domestic demand for similar brands in Estonia (Liivimaa lihaveis, http://liivimaalihaveis.ee and Muhu Liha http://muhuliha.ee) remains small, and the majority of calves from suckler herds on semi-natural pastures are sold abroad for rearing and consumption as a mainstream product (Külvet, pers. comm.). In Finland, a certification prototype (Luonnonlaidunliha) remains stillborn due to the small number of interested producers and a lack of organisational support for mainstreaming and supervising the proposed label. No such brand exists to date in Latvia, Lithuania or Norway, though some farmers sell their products directly to consumers by specifically marketing the use of semi-natural pastures (Birge 2019).

The contrasts in public awareness among the countries may be rooted in differing contexts. Semi-natural habitats are relatively common in rural landscapes of Sweden, and Swedish people acknowledge them as a cultural asset. However, as exemplified by the Finnish case, the extreme rarity of semi-natural habitats may erase this type of land use from the public mindscape. The latter could indicate a shifting baseline that drives our understanding and perception of landscapes and human-nature relationship (Soga and Gaston 2018). In Estonia, a relative ubiquity of abandoned semi-natural grasslands may set back their appreciation for conservation or heritage among urban consumers. We suggest therefore that it is the *area under appropriate management* that best supports the social values attached to semi-natural habitats; focusing either on the coverage or tailored management is insufficient. Exploration of this claim calls for a cross-border research to probe links between public awareness on semi-natural habitats and, for example, brand-based purchasing decisions, which can increase the relevance of semi-natural habitats in production, farm economy and producer decision-making, and support the direly needed management. Modern data sources, such as social media, may provide novel evidence. Further focus could be on specific issues that are known or remain obscure to people of different reference groups, for example, rural vs. urban populations.

## Implications

We upscaled a central concept in conservation biology—the extinction vortex—to semi-natural habitats as socio-ecological systems and reviewed evidence on social processes that we believe belong to the potential socio-ecological extinction vortex in the European boreal region. We demonstrate that (i) the evidence for the existence of key social processes exists, though not complete and consistent for all countries, and (ii) the evidence for the dynamic interactions, which link the key social processes and maintain the socio-ecological extinction vortex, is patchy and often anecdotal. Ascertaining the existence of the socio-ecological extinction vortex needs a more focused research effort. The gap in understanding the role of professional education in shaping the professional skills for management, and attention in research, policy and development, seems to be particularly glaring.

The collected evidence shows that two of the proposed vortex loops have been counteracted in the boreal region: the public payments, research and political targets for conservation and restoration of semi-natural habitats have been promoted in all studied countries (loop 2) and the level of public awareness has risen at least in Sweden (loop 4). However, even in Sweden, the changes remain separate from each other and insufficient to turn the “area under appropriate management” onto a positive track nationally. Eriksson (2016) described how semi-natural habitats are reinterpreted in modern Swedish society “to become values associated with beauty and heritage and species' intrinsic values”. Yet, it seems that such non-utilitarian motivators for conservation-oriented programmes are not able to prevent the overall deterioration of semi-natural habitats. Managing the habitat remnants exclusively for their historic and educational values (‘museum landscapes scenario’ in Lomba et al. 2019) makes them “effectively extinct” as socio-ecological systems due to decoupling from contemporary agriculture.

Based on our understanding of the historic and current state of semi-natural habitats in the boreal Europe, we argue that at present only by addressing most, if not all, of the feedback processes—both from the societal viewpoint (e.g., generation of producer benefits, public awareness) and from the population ecological viewpoint (e.g., maintaining genetic diversity, increasing population sizes and connectivity)—can the vortex be truly arrested. A recent example from Estonia demonstrates the importance of targeting most of the loops in an integrated manner to reverse the phenomenon of a socio-ecological vortex we propose. By re-connecting production values with public benefits through branding, public payments, research and public awareness, a previously deteriorating trend in the western part of the country has been reversed to the degree that local farmers compete for access to large and well-accessible semi-natural grasslands eligible for public payments (Helm, pers comm.).

Semi-natural habitats may thus regain their importance as sites of quintessential sustainable multifunctional land use. The “back to the future” scenario (sensu Lomba et al. 2019) for enhancing a socio-ecological viability of HNV farmlands highlighted the core requirements: increasing societal recognition of multiple ecosystem services, adopting new paradigms in public interventions, empowering farmers and rural communities, fostering technological innovation and promoting multifunctional landscapes. As remarked by Röös et al. (2016), this should also put back the primary benefits of ruminants in food production as converters of biomass non-edible to humans and providers of fertility for arable land, thus avoiding food and feed competition and operating within the environmental limitations of the land.

We do not imply a perfect correspondence between population ecological and social processes, or species and habitat levels. Analogies could be useful in exploring a phenomenon in new light as, for example, in analogies between interactions of genomic components and those of species (Le Rouzic et al. 2007), or between ecological and economic systems (Pilinkiene and Mačiulis 2014). A socio-ecological system framework itself has been developed to combine relatively independent ecological and social subsystems that have functions and structures of their own, but affect each other (McGinnis and Ostrom 2014). The proposed socio-ecological extinction vortex framework has a potential to pave the way for a range of multi- and cross-disciplinary research directions, as well as developing sufficiently inclusive multi-pronged solutions. It could possibly be applied to other habitat types subjected to systematic human disturbance, such as those in urban environments, or in need of restoration.

Box 1: Main EU funding instruments for research, innovation and development of relevance to agriculture and conservation The LIFE Programme is the EU’s funding instrument for the environment and climate action. It was created in 1992 and includes Nature and Biodiversity among its four focus areas

The European Innovation Partnership for Agricultural productivity and Sustainability (EIP-AGRI) was launched in 2012. It brings together innovation actors (farmers, advisers, researchers, businesses, NGOs and others) in agriculture and forestry at the EU level in multi-actor projects and networks (funded under Horizon 2020) and operational groups (funded under the Rural Development Programmes)

The LEADER programme aims to “engage local actors in the design and delivery of strategies, decision-making and resource allocation for the development of their rural areas”. Established in 2008, it became the most important tool for engaging multiple actors around locally and regionally important rural issues. It is funded under the Rural Development Programmes

The Interreg Baltic Sea Region Programme operates in the EU Member States, with Norway as a partner country. It supports “integrated territorial development and cooperation for a more innovative, better accessible and sustainable Baltic Sea region”, thus having an even higher potential impact with larger budgets

Erasmus is an EU programme that covers education, training, youth and sport. It aims to "encourage collaboration and extension the universes of training and work"
